# Interaction-tailored organization of large-area colloidal assemblies

**DOI:** 10.3762/bjnano.9.150

**Published:** 2018-05-29

**Authors:** Silvia Rizzato, Elisabetta Primiceri, Anna Grazia Monteduro, Adriano Colombelli, Angelo Leo, Maria Grazia Manera, Roberto Rella, Giuseppe Maruccio

**Affiliations:** 1 Department of Mathematics and Physics "Ennio De Giorgi", Università del Salento, Via per Arnesano, Lecce, Italy; 2 CNR NANOTEC - Institute of Nanotechnology, Campus Ecotekne, Via Monteroni, Lecce, Italy; 3 National Institute of Gastroenterology “S. De Bellis” Research Hospital, via Turi 27, 70013, Castellana Grotte (Bari), Italy; 4 Institute for Microelectronics and Microsystems, IMM-CNR, Lecce, Italy

**Keywords:** colloidal lithography, electrostatic interactions, large-area nanostructure patterning, localized surface plasmon resonance, spherical nanoparticles

## Abstract

Colloidal lithography is an innovative fabrication technique employing spherical, nanoscale crystals as a lithographic mask for the low cost realization of nanoscale patterning. The features of the resulting nanostructures are related to the particle size, deposition conditions and interactions involved. In this work, we studied the absorption of polystyrene spheres onto a substrate and discuss the effect of particle–substrate and particle–particle interactions on their organization. Depending on the nature and the strength of the interactions acting in the colloidal film formation, two different strategies were developed in order to control the number of particles on the surface and the interparticle distance, namely changing the salt concentration and absorption time in the particle solution. These approaches enabled the realization of large area (≈cm^2^) patterning of nanoscale holes (nanoholes) and nanoscale disks (nanodisks) of different sizes and materials.

## Introduction

In recent years, ordered nanostructured arrays have attracted great interest because of their applications in many fields such as photonics/plasmonics [1], phononics [2–3], spintronics/magnonics [4–5], biosensors and energy harvesting [6–8]. For example, metal nanostructured systems, stimulated by incident light of a specific wavelength, can support localized surface plasmon resonant modes. The high sensitivity of these oscillations to refractive index changes in the surrounding environment was exploited for monitoring binding events in real time and detecting gas [9–10], protein–ligand interactions, nucleic acid and protein conformational changes [11–12]. On the other hand, magnonic [13–14] and phononic crystals [3,15] have attracted large interest for achieving control on spin and acoustic wave propagation and engineering of their band structures. Nanoparticle size, density, distribution, and interparticle distance are key parameters in the study of the above-mentioned phenomena; this is why a proper fabrication tool, able to guarantee good flexibility, is required to meet research goals.

Typically, the fabrication of nanostructured systems involves techniques such as electron beam and focused ion beam lithography in order to realize arrays of nanoscale features with precise size, shape and distribution control. However, these processes have high cost and low speed and these limitations encourage the development of alternative methods for parallel nanofabrication. Among them, colloidal lithography is emerging as an innovative strategy for fast and inexpensive realization of nanoscale patterns such as nanoscale holes (nanoholes), nanoscale disks (nanodisks), nanoscale dots (nanodots) and nanoscale rings (nanorings), combining the advantages of both top-down and bottom-up approaches [16–18]. In this technique, monodisperse, spherical, nanoscale materials (usually polystyrene nanoparticles) are self-assembled onto a substrate to form a 2D crystal used as a lithographic (positive or negative) mask. The size, shape and interspacing can be controlled on the nanoscale by changing the particle diameter as well as the conditions of film deposition. Indeed, the colloidal crystal array can be assembled by means of various approaches with their own advantages and limitations [19–21]. Closed-packed arrangements of the colloidal spheres have been demonstrated by spin coating, controlled evaporation, Langmuir–Blodgett coating or electrophoretic deposition techniques [18]. These strategies can enable the highly controlled fabrication of metal nanostructures over a very large area, allowing the realization of plasmonic materials characterized by tunable optical features [22].

Similar methods can be developed for the realization of non-close-packed distributions of colloidal particles, providing the proper flexibility to realize different kinds of nanostructures such as nanodisks or nanohole arrays for several applications [20–21,23]. To obtain particles that are regularly distributed on the substrate with a well-defined, tunable distance and size, a good knowledge and control is required regarding the nature and strength of the interactions involved in the colloidal film formation.

In this work, we report on a systematic investigation of the effect of particle–substrate and particle–particle interactions on the formation of ordered colloidal assemblies. To obtain good surface coverage, we exploited electrostatic interactions between negatively charged, polystyrene, spherical, nanoscale materials and a functionalized, positively charged surface. In the case of small spheres (diameter of 80 nm), the interparticle distance and the number of randomly adsorbed spheres on the surface were tuned by changing the ionic strength of the particle suspension as a means to adjust the strength of their electrostatic interactions. In the case of larger diameter spheres (500 nm), this approach presents some limitations. For these samples, while drying, the higher surface lateral capillary forces tend to induce aggregation and disorder. Nevertheless, we were able to control the surface coverage by tuning the absorption time in order to achieve a long range order.

Notably, we showed how this method can be easily transferred for use with different materials to produce large-area nanostructure arrays with controlled size and shape for application in localized surface plasmon resonance (LSPR) sensing and magnonics.

## Experimental

### Materials

Glass substrates (3.5 × 2.5 cm^2^) were obtained from Electro Optical Technologies. Polystyrene spheres with diameter of 80 ± 7 nm (sulfate latex with surface charge density 1.2 µC/cm^2^ and concentration 8% w/v) and 500 ± 50 nm (non-functionalized Polybeads^®^ – microspheres containing a slight anionic charge from sulfate ester, concentration 2.5% w/v) were respectively purchased from Invitrogen and Polyscience, Inc. Poly(diallyldimethylammonium chloride) (PDDA, MW 200000–350000), poly(sodium 4-styrenesulfonate) (PSS, MW 70000) and poly(allylamine hydrochloride) (PAH, MW 50000) were purchased from Sigma-Aldrich.

### Methods

**Figure 1:**
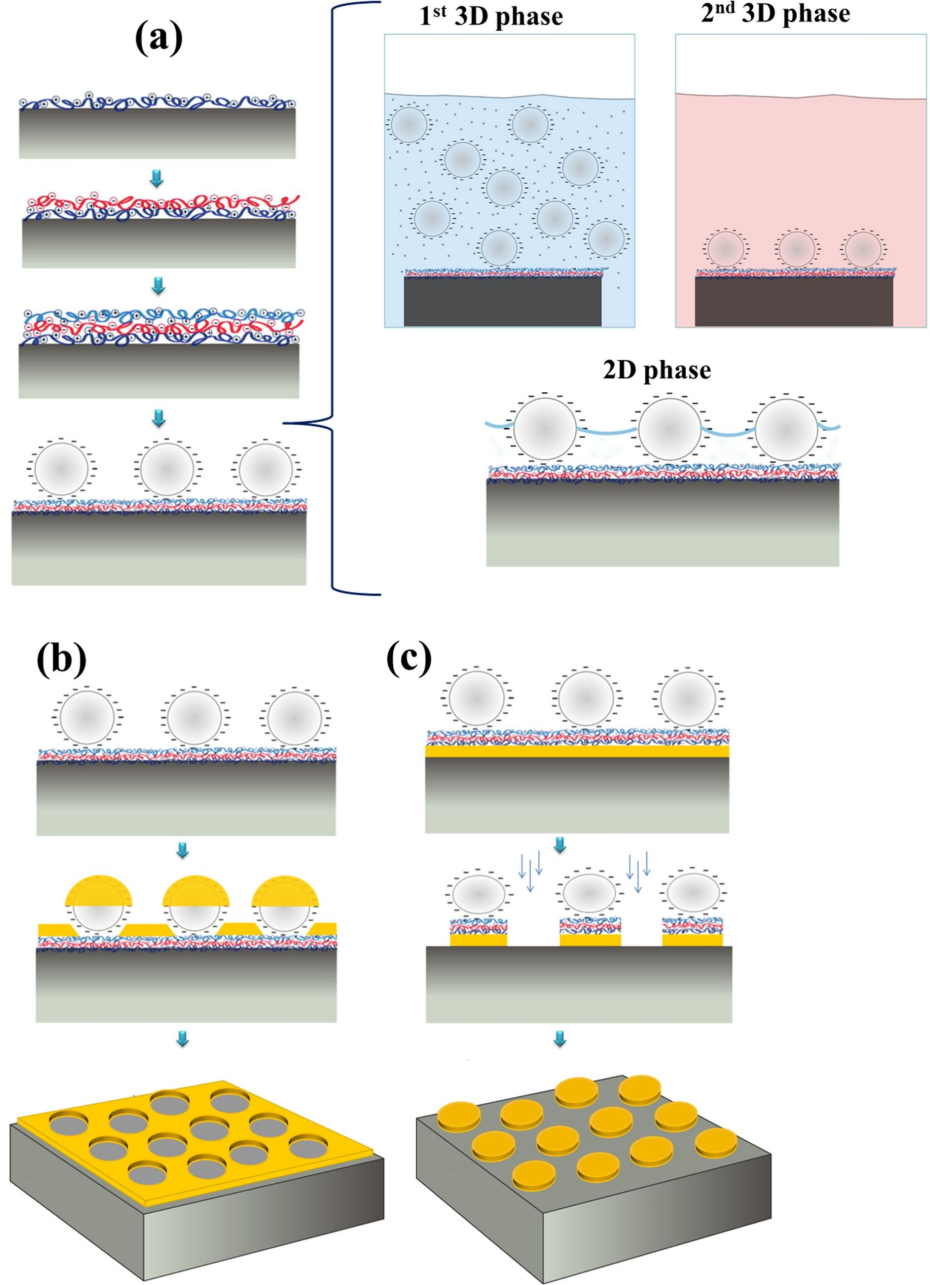
(a) Schematic drawing of the basic steps for the realization of a mask derived from spherical, nanoscale materials by electrostatic self-assembly of nanospheres for the fabrication of (b) nanoholes (c) and nanodisks.

The substrates were cut with a diamond tip into squares with an area of about 1 cm^2^ and cleaned in acetone and isopropanol. Given that the particles were negatively charged, the substrates were then functionalized to expose a positively charged surface in order to facilitate particle absorption by electrostatic interactions. For this purpose, the substrates were coated with a layer of 0.1 wt % PDDA, followed by 0.1 wt % PSS and 2 wt % PAH by alternately dipping into the respective aqueous solutions for 5 min with intermediate rinses in distilled water (Figure 1a).

Successively, the polystyrene spheres were assembled both onto bare (as reference) and functionalized substrates in a three-phase process. Initially, the samples were immersed in the suspension of spherical, nanoscale material to uniformly cover the substrate by allowing incubation from 10 min up to 24 h in the first (3D) assembly phase. Then, in the second phase, the samples were rinsed in milli-Q water to eliminate the excess of particles and placed in another beaker of milli-Q water at 100 °C for 60 s in order to increase the particle contact area on the substrate surface (and thus the adhesion forces) for preserving the electrostatically ordered configuration in the next steps [24]. Finally, the samples were rinsed again with milli-Q water at room temperature and carefully dried in a nitrogen flow; in this third (2D) assembly phase, however, (unwanted) lateral capillary forces could become relevant when the solvent film thickness approaches the particle diameter, as discussed later. As process parameters, the NaCl concentration and particle concentration in the assembly solution were tuned. The colloidal templates/samples fabricated are listed in Table 1 with relevant experimental details. Henceforward, we will use the following notation to refer to the samples: 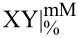, where X = B or F, indicating bare or functionalized substrates, respectively, Y = S or L, indicating small or large spheres, respectively, upper index refers to the NaCl concentration, and the lower index indicates the particle concentration in wt %.

**Table 1:** Colloidal film samples prepared in this work, given with relevant experimental details.

Sample/template	Diameter (nm)	Particle concentration (wt %)	NaCl concentration (mM)	Functionalization	Absorption time (min)	Sample code 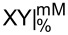

1	80	0.2	0	none	10	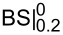
2	80	0.2	0	PDDA/PSS/PAH	10	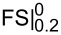
3	80	0.2	1	PDDA/PSS/PAH	10	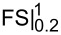
4	80	0.2	2	PDDA/PSS/PAH	10	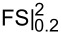
5	80	0.2	5	PDDA/PSS/PAH	10	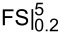
6	80	0.2	10	PDDA/PSS/PAH	10	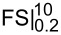
7	80	0.2	2	PDDA/PSS/PAH	60	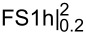
8	80	0.4	1	PDDA/PSS/PAH	10	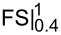
9	80	0.4	2	PDDA/PSS/PAH	10	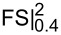
10	80	0.4	10	PDDA/PSS/PAH	10	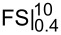
11	500	2	0	PDDA/PSS/PAH	10	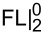
12	500	2	1	PDDA/PSS/PAH	10	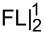
13	500	2	2	PDDA/PSS/PAH	10	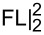
14	500	2	10	PDDA/PSS/PAH	10	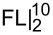
15	500	5	0	PDDA/PSS/PAH	10	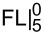
16	500	5	1	PDDA/PSS/PAH	10	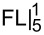
17	500	5	2	PDDA/PSS/PAH	10	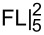
18	500	5	10	PDDA/PSS/PAH	10	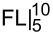
19	500	5	0	PDDA/PSS/PAH	60	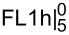
20	500	5	0	PDDA/PSS/PAH	1440 (24 h)	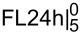

After colloidal assembly of the polystyrene particles, gold and cobalt nanohole arrays (Figure 1b) were realized by depositing 20 nm thick metal layers (gold films deposited by thermal evaporation and cobalt films deposited by magnetron sputtering) and successively removing the metal-capped spherical, nanoscale materials (termed “nanospheres” throughout the remainder of this article) by careful tape stripping with carbon tape in order to leave an array of ordered holes. In order to prepare the nanodisk lattices (Figure 1c), a gold film (20 and 40 nm thick films for particles of 80 nm and 500 nm diameter, respectively) was thermally evaporated on the glass substrate before layer-by-layer (LbL) deposition and colloidal assembly. Successively, reactive ion etching was used to selectively remove the portion of the gold film not protected by the nanospheres. The etch rate (2.9 nm/min) was estimated measuring the thickness of the gold film for different etching times. Finally, the nanosphere residues were removed by oxygen plasma treatment for 90 s, revealing the fabricated nanostructures on the substrates.

The morphology of the obtained patterns was imaged by means of a scanning electron microscope (SEM, Carl Zeiss) at an accelerating voltage of 5 kV. SEM images were acquired through an in-lens detector for secondary electrons in top-view configuration.

In order to analyze the particle distribution, ImageJ 1.42R (National Institutes of Health, USA) software was used and the images were processed to allow easier recognition. From each image, the background was eliminated and a threshold level was defined to identify the particle edges. Then the particles were filled in order to extract the coverage area (area of particles divided by total area), which was calculated for each condition/sample considering the average of at least three images from different areas. The coordinates of the particle centers were also acquired and used to compute the radial distribution function, which permits estimate of the interparticle distance and is defined as *g*(*r*) = ρ(*r*) / ρ_0_ where ρ(*r*) is the average concentration of particle centers in a circular shell with radius *r* around a particle and ρ_0_ is the average concentration of particles on the whole surface considered.

The optical absorbance of the fabricated nanostructure was characterized by a Cary500 UV–visible spectrophotometer. All the spectra were taken in the vis–NIR spectral range at room temperature and compared with expected theoretical results obtained through numerical modelling. A simple 3D model based on finite element analysis was implemented in Comsol Multiphysics software in order to investigate the optical response of single and ordered arrays of different gold nanostructures under the excitation of a uniform p-polarized electromagnetic field.

The magnetic properties of cobalt nanoholes were investigated by the magneto-optical Kerr effect technique in longitudinal configuration. The samples were placed between the poles of a GMW 3470 electromagnet, where the magnetic field intensity was measured by a Group3 Teslameter probe. A He–Ne laser beam (wavelength 633 nm, radiation power 4 mW) was polarized and modulated at 173 Hz using a mechanical chopper. The reflected beam was analyzed by a second polarizer and collected by a Si-biased detector (Thorlabs, DET10A/M). The output signal was acquired by a Stanford Research SR830 lock-in amplifier tuned at the chopper frequency. The angle between the incident and reflected light beams was set at 30°.

## Results and Discussion

**Figure 2:**
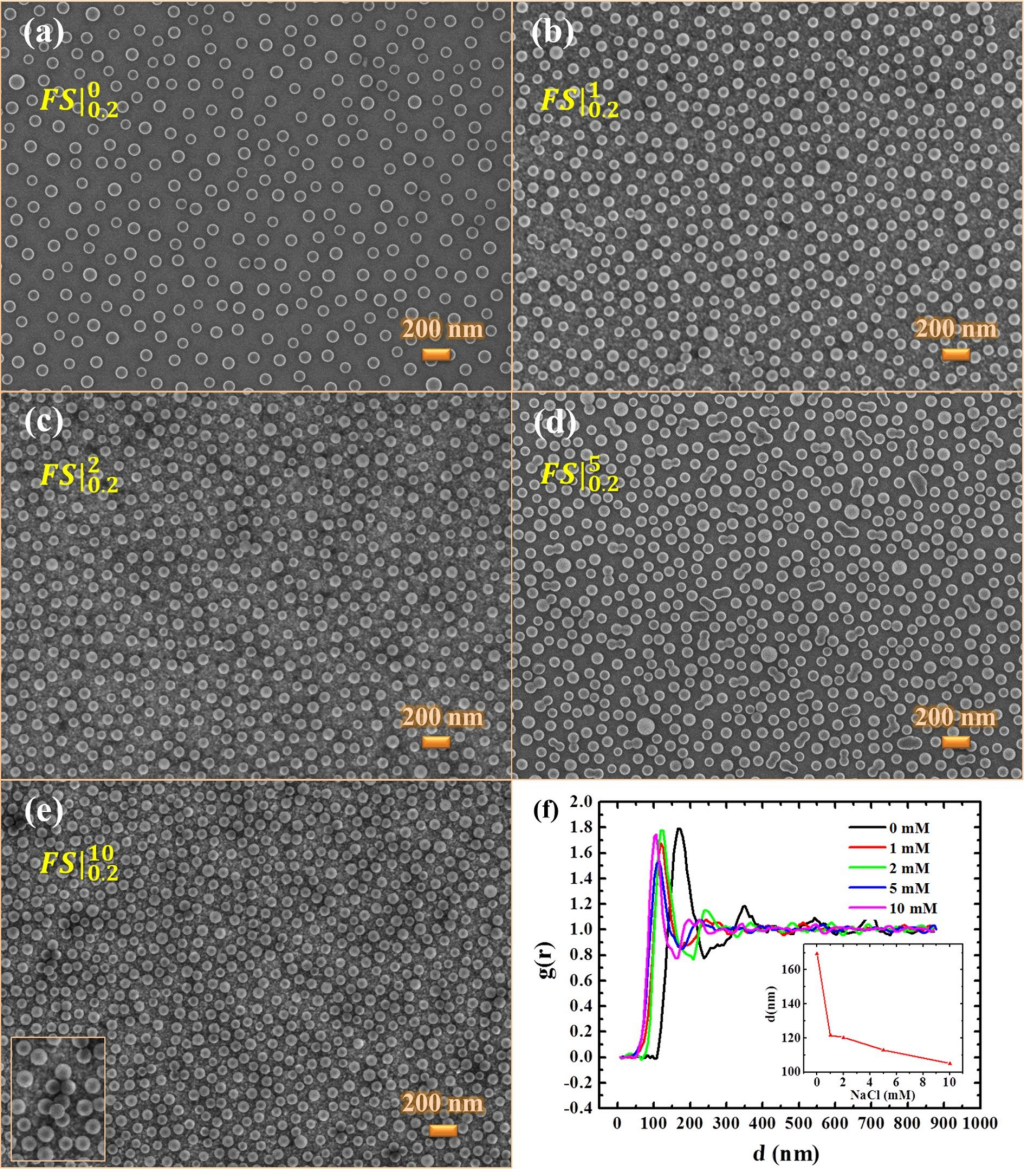
SEM plane-view of nanosphere masks obtained by solutions with different salt concentrations: (a) 0 mM (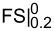), (b) 1 mM (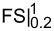), (c) 2 mM (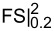), (d) 5 mM (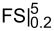), (e) 10 mM (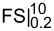). The particle concentration was 0.2 wt %, the absorption time was 10 min and the diameter of the particles was 80 nm. (f) Radial distribution function and (inset) average interparticle distance as a function of salt concentration.

The effect of particle–substrate interactions on colloidal self-assembly was the first aspect that was investigated. For this purpose, the charged polystyrene spheres of smaller diameter were deposited both on bare and positively charged functionalized substrates. In the case of bare substrates (samples labeled as BS), only few, rather isolated particles were observed on the surface. We ascribed these results to a poor adhesion between the particles and the substrate, leading the particles to spread out during the rising process. The functionalized substrates (samples labeled as FS) allow for improved particle anchorage on surfaces modified by the absorption of three oppositely charged polyelectrolytes. In this case, the attraction exerted by the substrate combined with the repulsive interactions between the negatively charged particles led to the formation of a relatively ordered colloid pattern (as shown in the SEM image in Figure 2a) covering the entire functionalized surface.

Then, particle–particle interaction effects were investigated for tailoring the ordered nanosphere arrays. In an electrolyte solution, the particles interact at a sufficiently large distance *r* (greater than the particle radius) through a screened Coulomb potential *u*(*r*) 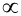 e^−κ^*^r^* / *r* [25–26]. The range of particle electrostatic repulsion is determined by the Debye length 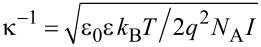 where *q* is the elementary charge, *N*_A_ is Avogadro’s number, *k*_B_ is Boltzmann’s constant, *T* is the absolute temperature and 
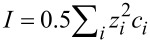
 is the ionic strength whereby the electrolyte solution contains ion of type *i* with valence *z_i_* and molar concentration *c_i_*. Therefore, the strength of electrostatic interaction between the colloidal particles on the surface can be controlled by varying the salt concentration in the nanosphere suspension during the first 3D assembly phase. In this way, different samples (samples 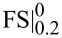, 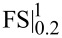, 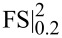, 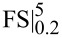 and 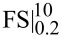) were prepared using sulfate-modified negatively charged polystyrene spheres with diameter of 80 nm in solutions with different salt (NaCl) concentration (from 0 mM to 10 mM).

Figure 2a–e reports SEM images of 80 nm particle films obtained by absorbing solutions with different salt concentrations but at the same 0.2 wt % nanosphere concentration, while the relative radial distribution functions *g*(*r*) are compared in Figure 2f. The clear primary peak in the *g*(*r*) function, whose *x*-coordinate corresponds to the nearest center-to-center mean distance, suggests the presence of short range ordering. On the other hand, the constant value at longer distances indicates the absence of a long range order. As shown in the inset of Figure 2f, the salt concentration influences the distance between the particles, and consequently, the surface coverage. For 80 nm polystyrene spheres, the coverage increases from 18% without NaCl to 38% for 10 mM salt concentration (Table 2), as a consequence of the decreasing particle distance. Indeed, the electrostatic repulsion between the particles is more screened by an increased electrolyte concentration, which allows them to adsorb more closely and achieves a higher surface coverage. At 5 mM salt concentration, the particles are so close that doublet and triplet aggregates begin to form on the surface, whereas clusters of more than three particles are visible at 10 mM (inset of Figure 2e).

**Table 2:** Coverage as a function of particle diameter/concentration and salt concentration. The absorption time was 10 min in all cases.

Diameter (nm)	Particle concentration (wt %)	NaCl concentration (mM)	Coverage (%)

80	0.2	0	18
		1	27
		2	27
		5	33
		10	38
80	0.4	1	27
		2	27
		10	39
500	2	0	33
		1	42
		2	46
		10	48
500	5	0	36
		1	44
		2	45
		10	48

The same study was repeated using an increased particle concentration in the solutions (sample 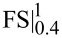, 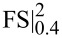, and 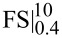) but the obtained substrate coverage was apparently not significantly affected, at least in the investigated range (variations of 1–2% were found from 0.2 to 0.4 wt % particle concentration). Moreover, a negligible influence of the absorption time on the coverage was observed when the suspension was left on the substrate for 1 h compared to 10 min (sample 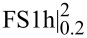). We conclude that no more particles can be accommodated in the assembly once the maximum packing limit is obtained

The nanosphere template formation can be better understood if we analyze the dynamics of the assembly process in its three phases more in depth. In the first 3D assembly phase, when the substrate is totally immersed in the nanosphere suspension, the particles diffuse in the solution and electrostatically adsorb one by one on the surface, as predicted by random sequential absorption (RSA) theory. According to this model, the new particles do not overlap with particles already fixed on the substrate, but rather regularly absorb with a certain separation from their neighbors, depending on the interparticle repulsion intensity (which is related to the salt concentration). Once absorbed, it cannot be moved. This “irreversible” absorption process continues until a packing limit ρ_max_, is reached, where the surface density of the nanosphere saturates, which is related to the ionic strength of the nanosphere suspension [24,26–27]. For this reason, as seen experimentally, both the particle concentration and time absorption do not significantly affect the coverage.

Then, during the second phase, the rising of the sample in milli-Q water allows removal of the particles not properly absorbed to the substrate by electrostatic interaction. Immersing the still wet sample in a boiling water bath allows preservation of the electrostatically assembled, ordered structure by increasing the particle contact area (and adhesion) on the substrate surface [24]. In the third 2D assembly phase, during the unavoidable drying process, when the solvent film thickness reaches the particle diameter, unwanted attractive lateral capillary forces [24,28] can become relevant. This results in particle displacements and deviations from the RSA model since the particle are not completely fixed in their positions on the substrates [29].

It is worth noting that the intensity of capillary forces increases with the particle radius and as the distance between two particles in a liquid is reduced: *F*_cap_ ≈ 2πσ*r*_c_^2^(sin^2^θ) / *r* where σ is the surface tension of the liquid, θ is the mean meniscus slope angle at the contact line, *r*_c_^2^ is the radius of the three phase contact line at the particle surface and is related to the particle radius, and *r* is the distance between the centers of the particles [30–31]. Thus, their influence is not very evident in the case of small and well-separated particles (Figure 2a). On the other hand, their contribution increases at higher ionic concentration when the interparticle gap becomes shorter. In this case, capillary forces can exceed electrostatic interactions, tearing off some particles from their position in the regular 2D array and inducing the random formation of some small particle aggregates on the surface (on a small scale because of instability of thin water films, mainly for small particles ≈100 nm) [32–33].

**Figure 3:**
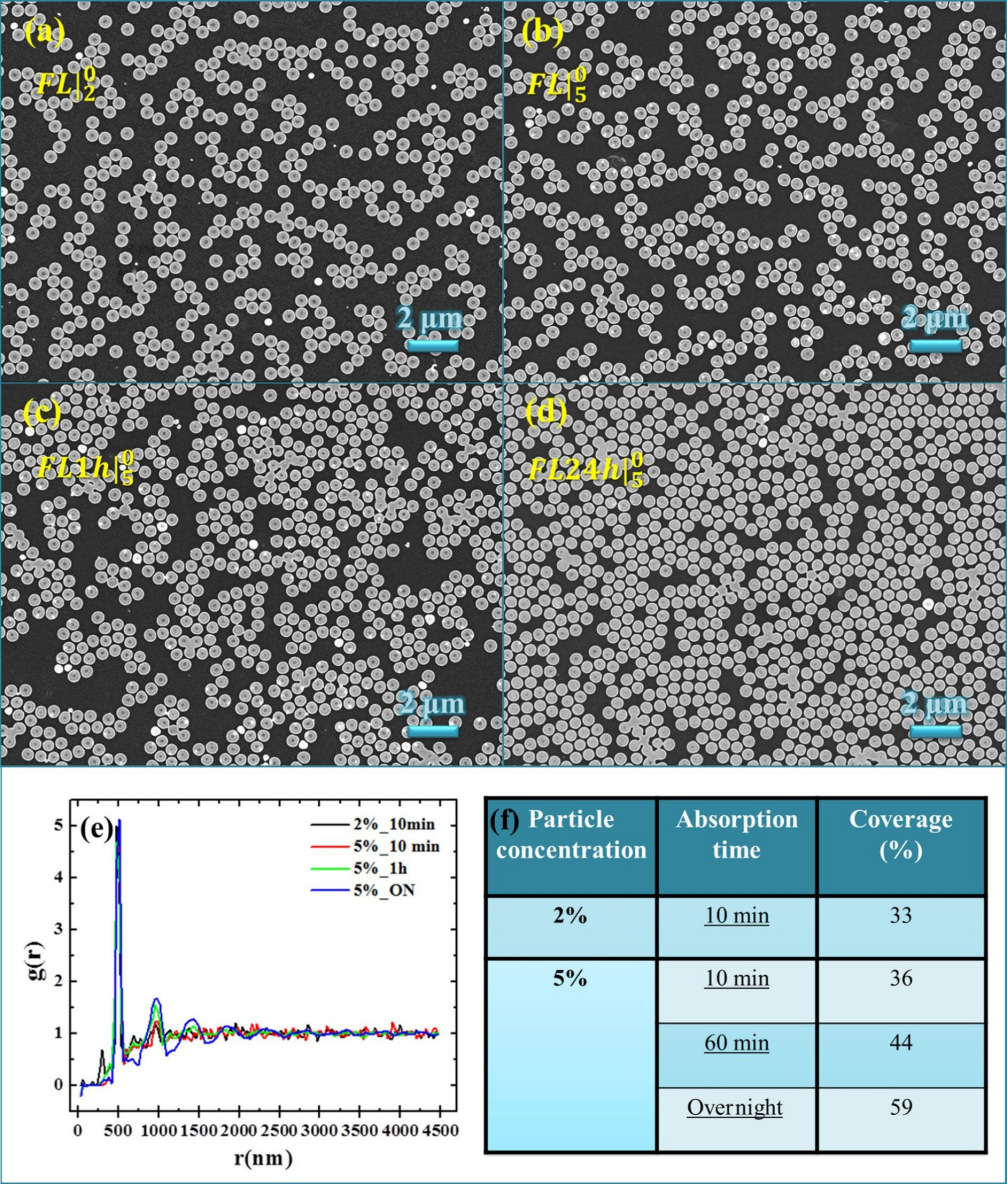
SEM plane-view of nanospheres masks obtained for different particle concentrations and absorption times: (a) 2 wt % for 10 min (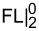), (b) 5 wt % for 10 min (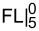), (c) 5 wt % for 1 h (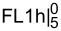), (d) 5 wt % overnight (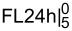). (e) Radial distribution function for different absorption times. In (f) Coverage obtained for different particle concentrations and absorption times. The diameter of the particles was 500 nm in all cases. In the starting solution, the salt concentration was 0 mM.

In the case of particles with larger diameters, the nanosphere organization is different, since lateral capillary attraction gains strength relative to the interparticle repulsive and particle substrate adhesion forces. To investigate the assembly regime driven by lateral capillary forces, non-functionalized, lowly charged particles of 500 nm diameter were then employed. First, samples were prepared without salt using a 2 wt % particle concentration and 10 min as the absorption time (sample 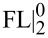). From SEM analysis of Figure 3a, a 33% coverage was calculated and the radial distribution function in Figure 3e was extracted. This distribution exhibits one peak at an interparticle distance of about 500 nm, which roughly corresponds to the particle diameter and indicates touching first neighbor particles. This kind of assembly can be ascribed to the high capillary forces acting during the solvent evaporation, which pull the particles together to form regions of closed-packed spheres, leaving voids on substrate. This effect was more evident with the 500 nm diameter particles since the capillary strength is proportional to the particle radius and the surface charge was much lower. When the particle concentration increases from 2 to 5 wt % (sample 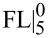), the resulting radial distribution function is very similar, despite a higher surface coverage (around 36%).

As the next step, the salt concentration was modified also for solutions of large particles (samples 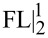, 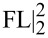 and 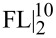). Table 2 shows how the coverage increased significantly when the NaCl concentration increased from 0 mM (sample 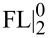) to 1 mM (sample 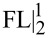), while minor variations were observed by further increasing the salt amount up to 10 mM (samples 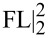 and 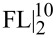) since the 500 nm particles present only a slight anionic charge. The same trend was exhibited at higher particle concentration (5 wt %, samples 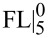, 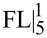, 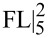 and 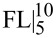). The absorption process under the influence of electrostatic particle–particle interactions was already applied in [18] but no coverage control with particles of 500 nm diameter was achieved because of high lateral capillary forces.

For increasing absorption times, at 5 wt % particle concentration without NaCl (samples 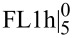 and 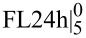), the number of particles that settle out of the solvent and adhere onto the substrate surface increases, and the coverage was observed to rise from 36% after 10 min to 44% after 60 min (sample 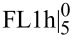) and 59% after overnight incubation (sample 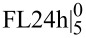) (Figure 3b–d and Figure 3f). The nanosphere distribution was also affected, resulting in the different radial distribution functions shown in Figure 3e; all the curves show one peak at a distance of about one particle diameter, which is due to particles stacking together. However, other well-defined peaks appear in *g*(*r*), increasing the time that corresponds to the preferred second, third neighbor distances and indicates the achievement of a long-range order.

Once the parameters required for the assembly of colloidal nanoparticles in regular 2D arrays were identified, their functional role as masks for the realization of metal nanostructure arrays on planar substrates was investigated, in view of their possible applications in plasmonic and magnonic fields. In both cases, nanoparticle size, distribution and nanostructure mesospacing are key parameters for the control of the electric or magnetic field distribution and intensity on the investigated area.

Concerning plasmonic applications, the excitation of localized surface plasmon resonance (LSPR) results in strong light scattering and absorption as well as enhanced electromagnetic fields in proximity of the metal structures. These properties are strongly dependent on particles size, geometry and distribution. As an example, applications based on the enhancement of the absorption cross-section (ACS) require the fabrication of plasmonic nanoparticles characterized by relatively small dimensions (e.g. ≈40 nm). On the contrary, applications based on the strong enhancement of the scattering cross-section (SCS), are generally associated to the presence of larger nanoparticles (e.g. ≈80 nm) [34]*.* Another critical parameter affecting both the sensitivity and the electromagnetic field distribution around plasmonic nanostructures is the interparticle spacing. Higher wavelength sensitivity and larger sensing volume have been theoretically and experimentally demonstrated for nanostructured system characterized by greater average interparticle spacing, owing to the wider distribution of the electric field when the resonance conditions are satisfied [35–36].

**Figure 4:**
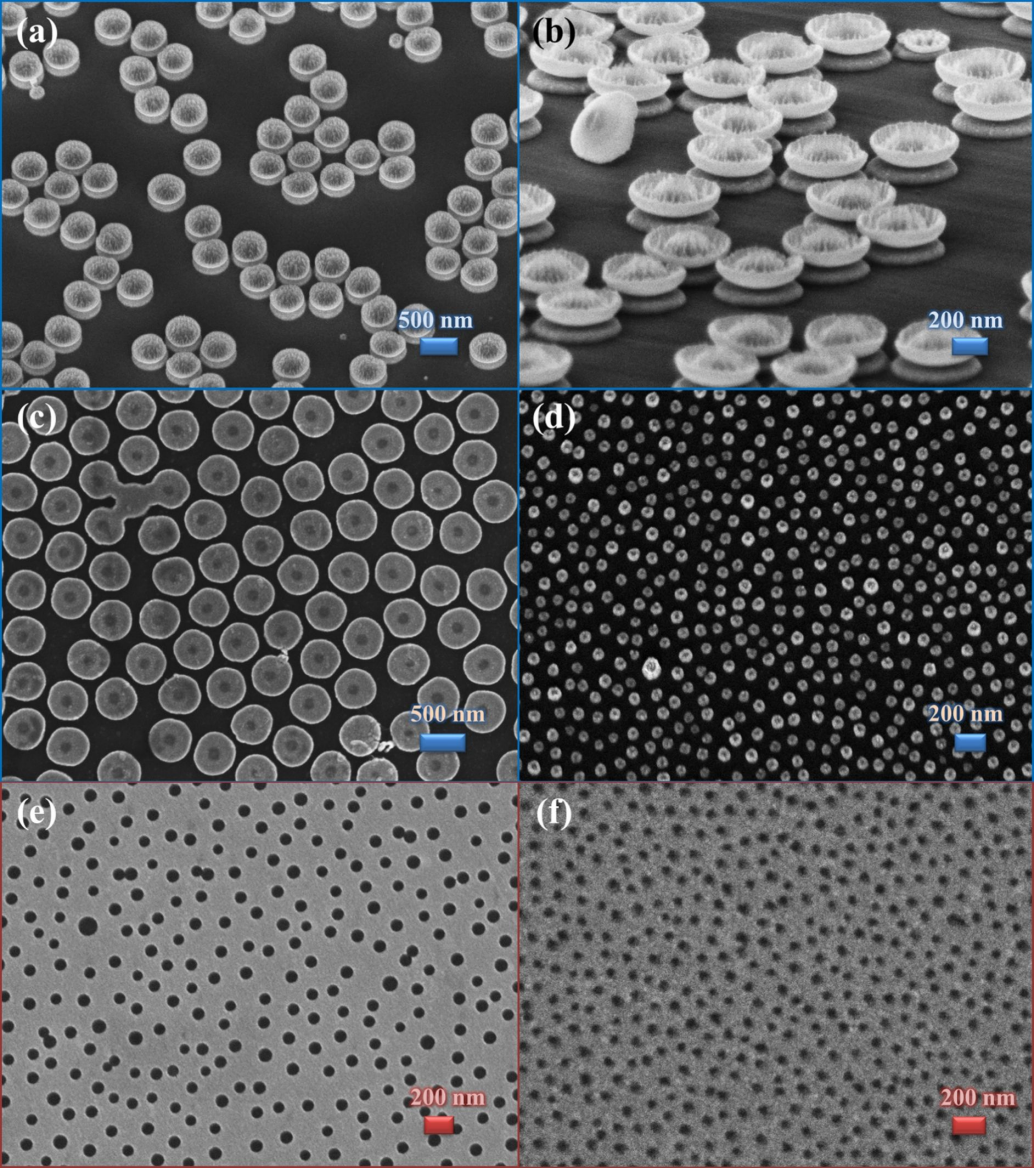
(a,b) Gold nanodisks covered by the etched nanospheres of 500 nm. (c,d) Gold nanodisks of different diameter and thickness. (e,f) Nanoholes in gold film and cobalt, respectively.

It appears clear that, for practical purposes, a proper compromise between size and average interparticle distance should be achieved. This ratio guided the choice and optimization in this work of the two strategies of colloidal assembly for 80 nm and 500 nm particles, which were exploited to create different types of nanoscale features with specific characteristics. From these assemblies, nanodisks and nanoholes of different sizes and materials were realized. Figure 4a,b shows gold nanodisks of 500 nm still covered by the nanosphere portion remaining after the etching process, while in Figure 4c,d images of the final disks of different diameters are reported. Gold and cobalt nanoholes realized by means of the same technique are shown in Figure 4e,f.

For the development of plasmonic materials characterized by sharp LSPR resonances in a desired spectral range, and for the interpretation of experimental measurements, the optical response of gold nanodisk and nanoholes realized on glass substrates has been calculated using a numerical model developed in COMSOL.

**Figure 5:**
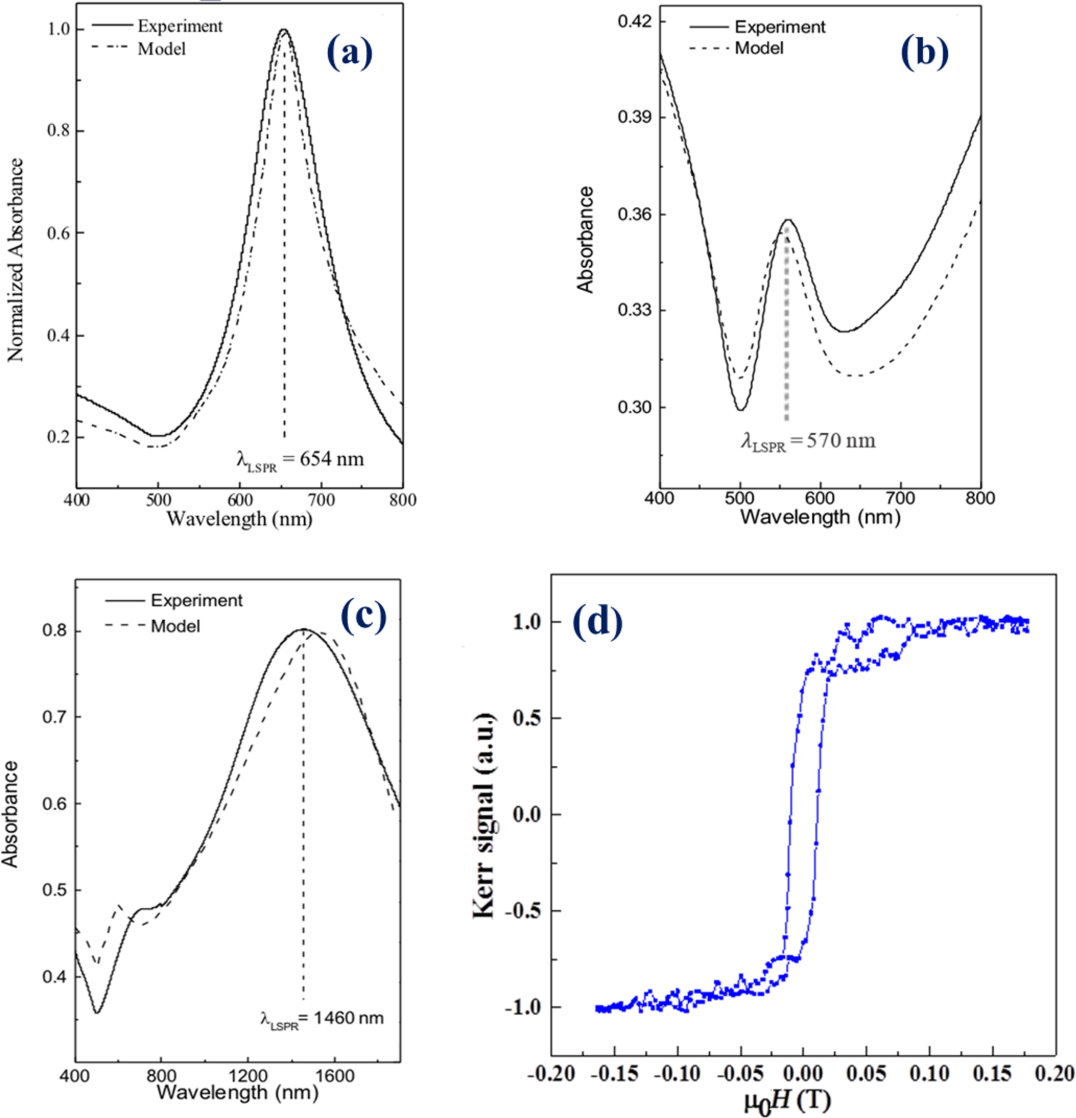
Absorbance spectra of (a) 80 nm gold nanodisk, (b) 80 nm gold nanoholes and (c) 500 nm gold nanodisk. All the spectra have been acquired at room temperature in the vis-NIR spectral range and compared with numerical results (dotted lines). (d) Longitudinal magneto-optic Kerr effect (MOKE) hysteresis loops of the 80 nm cobalt nanohole sample.

In Figure 5a,b, simulated curves are compared with experimental absorbance spectra of gold nanodisks and nanoholes distributed on glass substrates, respectively. For these samples, the features have an average diameter of 80 nm. Upon excitation of the LSPR, the absorbance signal of the fabricated nanostructures exhibits a pronounced peak in the visible spectral range. On the contrary, LSPR resonances in the infrared spectral range can be obtained by tuning the size distribution of the nanostructures towards 500 nm, as reported in Figure 5c. Evidently, these structures can find application in complementary fields of sensing applications, depending on the functional aspects to be investigated. A very good accordance with the expected theoretical results is demonstrated for both sets of nanostructures arrays, revealing that a theoretically driven route for a feasible fabrication of metal nanostructures has been demonstrated with this work.

Concerning magnetic samples and their properties, a hysteresis loop of cobalt nanoholes is presented in Figure 5d as obtained by magneto-optic Kerr effect (MOKE) measurements – a ferromagnetic behavior with saturation at high fields was clearly exhibited.

## Conclusion

Ordered nanostructured arrays are key elements for applications in various fields; however, methods for producing them over large areas and at low cost are needed for full exploitation.

In this manuscript, we showed that the organization of colloidal assemblies on the substrate depends on the particle–surface interaction but also by the forces exerted between the particles themselves during the particle absorption and drying processes. In the case of bare substrates, few particles were deposited on the surface because of the weak adhesion between the particles and the substrate. Instead, in the case of functionalized substrates, the electrostatic interactions between negatively charged polystyrene nanospheres and a positively charged surface allowed the formation of relatively ordered colloid patterns extended over a centimeter scale. In the case of small particles (diameter of 80 nm), tuning the strength of electrostatic interactions by tuning the NaCl concentration in the suspension was found to be an effective approach to control the interparticle distance and the coverage. On the other hand, for larger diameter spheres (500 nm), the higher capillary forces tend to prevail during the drying process and to tailor the number of deposited polystyrene spheres it was preferable to modify the absorption time. The colloidal assemblies resulting from the optimization of these two strategies were employed as lithography masks for the realization of nanoholes and nanodisks of different sizes and materials covering the whole functionalized substrate.

In conclusion, we reported a simple and flexible method for the production of relatively ordered nanoparticle patterns that can be employed on any material to realize nanostructures of tunable shape, size and distance. The latter can be varied according to the nature and strength of the interactions involved in the colloidal film formation. These results are relevant for applications where the realization of large area nanoscale features with tunable properties is required.
